# The contribution of second primary cancers to the mortality of patients with a first primary breast cancer

**DOI:** 10.1007/s10549-024-07361-3

**Published:** 2024-06-13

**Authors:** Elisabete Gonçalves, Filipa Fontes, Jéssica Rocha Rodrigues, Rita Calisto, Maria José Bento, Nuno Lunet, Samantha Morais

**Affiliations:** 1 EPIUnit – Instituto de Saúde Pública, Universidade do Porto, Rua das Taipas 135, 4050-091 Porto, Portugal; 2 Laboratório Para a Investigação Integrativa e Translacional em Saúde Populacional (ITR), Porto, Portugal; 3 Departamento de Ciências da Saúde Pública e Forenses e Educação Médica, Faculdade de Medicina da Universidade do Porto, Alameda Professor Hernâni Monteiro, 4200-319 Porto, Portugal; 4 Unidade de Investigação em Enfermagem Oncológica - Centro de Investigação (CI-IPOP) & Porto Comprehensive Cancer Center (Porto.CCC) & RISE@CI-IPOP (Rede de Investigação em Saúde), Instituto Português de Oncologia do Porto FG, EPE (IPO-Porto), Rua Dr. António Bernardino de Almeida 865, 4200-072 Porto, Portugal; 5 Grupo de Investigação em Epidemiologia, Resultados, Economia e Gestão em Oncologia - Centro de Investigação (CI-IPOP) & Porto Comprehensive Cancer Center (Porto.CCC) & RISE@CI-IPOP (Rede de Investigação em Saúde), Instituto Português de Oncologia do Porto FG, EPE (IPO-Porto), Porto, Portugal; 6 Serviço de Epidemiologia, Instituto Português de Oncologia do Porto FG, EPE (IPO-Porto), Porto, Portugal; 7 Departamento de Estudos de Populações, ICBAS - Instituto de Ciências Biomédicas Abel Salazar da Universidade do Porto, Rua de Jorge Viterbo Ferreira 228, 4050-313 Porto, Portugal

**Keywords:** Breast neoplasms, Epidemiology, Population register, Mortality, Second primary neoplasm

## Abstract

**Purpose:**

Second primary cancers (SPCs) are estimated to affect nearly 5% of patients with breast cancer within 10 years of their diagnosis. This study aimed to estimate the contribution of SPCs to the mortality of patients with a breast first primary cancer (FPC).

**Methods:**

A population-based cohort of 17,210 patients with a breast FPC diagnosed between 2000 and 2010 was followed for SPCs (31/12/2015) and vital status (30/06/2021). Patients diagnosed with an SPC (265 synchronous and 897 metachronous, ≤ 1 and > 1 year after the FPC, respectively) were matched (1:3, by five-year age group and year of breast FPC diagnosis) to those without an SPC and alive when the corresponding SPC was diagnosed.

**Results:**

Significantly higher hazards of death were found among patients with an SPC [hazard ratio of 1.56, 95% confidence interval (CI) 1.29–1.89 for synchronous SPCs; and 2.85, 95%CI 2.56–3.17 for metachronous SPCs] compared to patients with a breast FPC only. Estimates were higher for synchronous lung, stomach, non-Hodgkin lymphoma and breast SPCs, and metachronous liver, stomach, ovary, lung, rectum, corpus uteri, colon, breast, and non-Hodgkin lymphoma SPCs. The 15-year cumulative mortality was 59.5% for synchronous SPCs and 68.7% for metachronous SPCs, which was higher than in patients with a breast FPC only (43.6% and 44.8%, respectively).

**Conclusions:**

In Northern Portugal, patients with an SPC following a breast FPC have a higher mortality compared with patients with a breast FPC only.

**Supplementary Information:**

The online version contains supplementary material available at 10.1007/s10549-024-07361-3.

## Introduction

Breast cancer is the most incident cancer worldwide, accounting for 2.3 million new cancer cases in 2020. Further, the number of females living with a history of breast cancer in 2020 is estimated to be 7.8 million globally (five-year prevalence) [1, 2]. This occurs because of overall high survival, which reflects early diagnoses and improved therapies [3–5]. Survivors of breast cancer are at risk of adverse medical outcomes, such, as subsequent primary cancers, mainly in the breast, endometrium, ovary, thyroid, lung, soft tissue sarcomas, leukemia, melanoma, and digestive organs [6]. Moreover, patients with a breast cancer share common risk factors, such as genetic, environmental, and hormonal, for a second primary cancer (SPC), and may suffer from side effects of cancer therapy [7–9].

The diagnosis of an SPC may be a major event among patients with a breast first primary cancer (FPC) [8]; in Europe, SPCs are diagnosed among nearly 5% of survivors of breast cancer within 10 years [10]. Previous studies have suggested that these patients, when diagnosed with an SPC, may have a worse prognosis compared to patients without an SPC [11–14]. However, the methodological framework related to the mortality of patients with an SPC is difficult, due to the contribution of each cancer to death, as well as other non-oncological causes of death [15]. Some studies have focused only on breast SPCs after a breast FPC or were incorporated in multi-cancer studies [11–14].

In Northern Portugal, breast cancer is the most incident cancer, accounting for nearly one third of female tumors and it is the first cause of cancer-related death [16, 17]. A previous study conducted in Northern Portugal evaluating SPCs after any FPC, found the prognosis of patients diagnosed with an SPC shortly after an FPC primarily depended on the type of FPC [18]. In contrast, for patients diagnosed with an SPC after a prolonged period following the initial FPC, survival outcomes were influenced by the specific site of the SPC. Considering the overall high survival from breast cancer, this study aims to extend previous observations of the incidence of multiple primary cancers in a cohort of patients with a breast cancer [19, 20], by estimating the contribution of an SPC to the mortality of patients with a breast FPC.

## Methods

### Study setting

A cohort of patients with breast cancer was retrieved from the North Region Cancer Registry (RORENO). This is a population-based cancer registry established in 1988 that covers Northern Portugal, representing over three million residents [16]. All incident cancers occurring in the catchment area of the registry are recorded, either by public or private hospitals and clinics, and private pathology laboratories. Registration follows the International Agency for Research on Cancer (IARC) rules, which include four dimensions: comparability, validity, timeliness and completeness [21]. The quality of the registry is maintained through routine screening with pre-defined algorithms for consistency and validity.

### Definition of multiple primary cancers

Multiple primary cancers were defined as proposed by the International Association of Cancer Registries (IACR) and IARC [22]. Briefly, primary cancers are those that originally developed in an organ or tissue, not being an extension, recurrence, or metastasis. Different morphologies (even with the same topography) or dissimilar topographies should be regarded as multiple primary cancers, regardless of the time between diagnoses, unless they correspond to systemic cancers, which are considered the same cancer. Multiple primary cancers were defined as SPCs, when two primary cancers were diagnosed in the same individual, and third or higher order primary cancers when more than two primary cancers were registered.

### Study design

All primary invasive tumors of the breast [International Statistical Classification of Diseases and Related Health Problems 10th Revision [23] (code C50)] diagnosed among female residents in Northern Portugal between 1 January 2000 and 31 December 2010 were identified (n = 17,210). Patients who had a previous cancer diagnosis other than skin non-melanoma (n = 1064) were excluded.

Vital status (death by any cause) was assessed through the National Health Service database, up to 30 June 2021, and subjects who could not be linked to the National Health Service database (n = 165) were excluded. The latter were not significantly different from included participants regarding age at diagnosis of the breast FPC [median (percentile 25 – percentile 75): included – 57 (47–68) vs. excluded – 59 (48–76); p = 0.058].

The remaining patients (n = 15,981) were followed to 31 December 2015 to identify multiple primary cancers through record linkage with the list of cases registered by RORENO. Those diagnosed with third or higher order primary cancers (n = 67) were excluded [19]; thus, 1162 SPCs were available for the present study. SPCs were classified as synchronous when diagnosed within 12 months of the breast FPC (n = 265) or metachronous otherwise (n = 897), as previous studies on this topic [24].

**Fig. 1:**
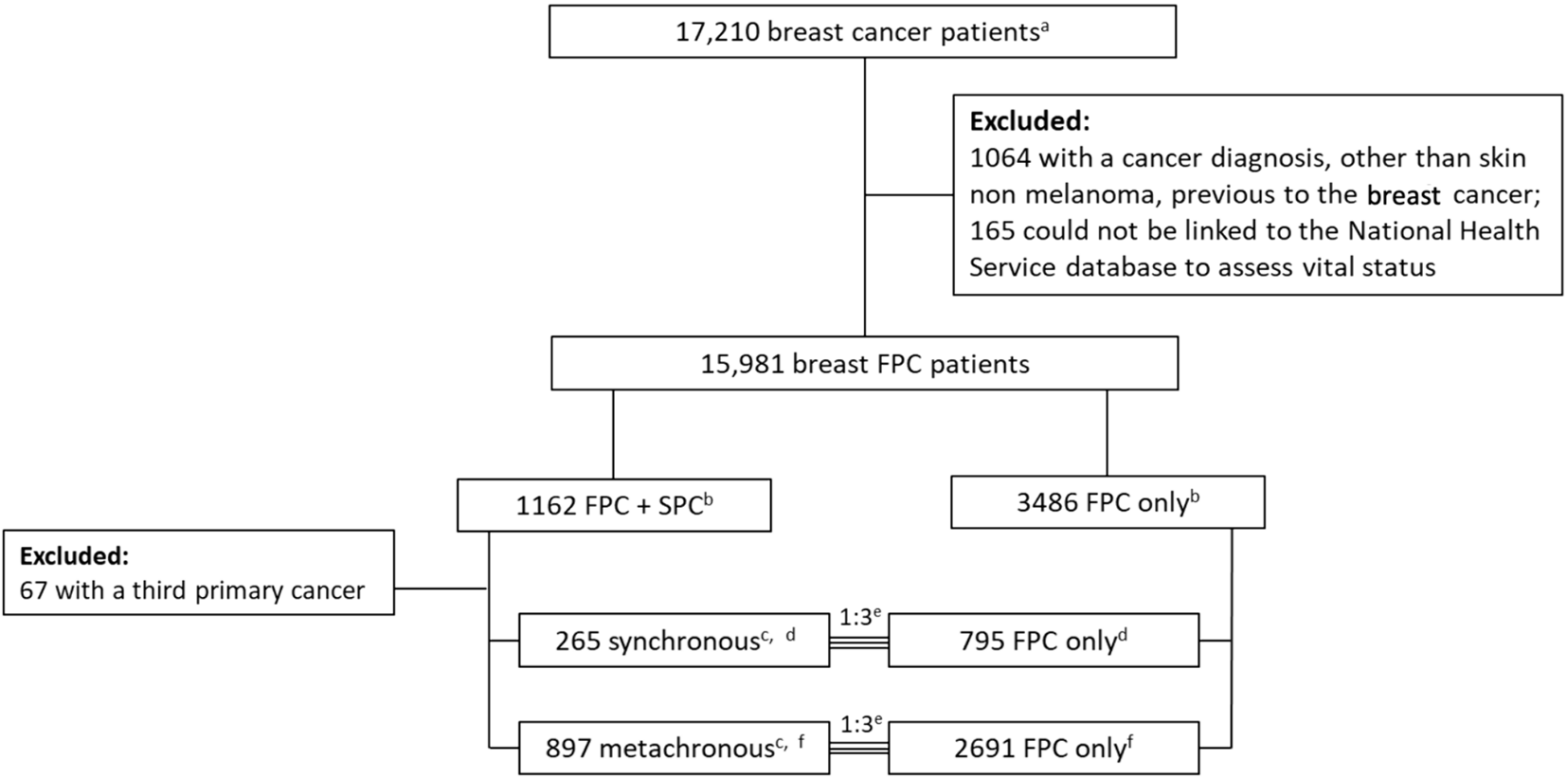
Flowchart of patient inclusion in the study. *FPC* first primary cancer, *SPC* second primary cancer. ^a^Diagnosed between 1 January 2000 and 31 December 2010. ^b^Follow-up to 30 June 2021. ^c^Synchronous when diagnosed within 12 months of the breast FPC and metachronous, otherwise; follow-up to 31 December 2015. ^d^The median (percentile 25—percentile 75) age at diagnosis of FPC was 61 (49–72) for synchronous SPC and 62 (49–73) for the corresponding FPC only patients. ^e^Matched 1:3 by five-year age group (from 15–19 to 70–74, and ≥ 75) at breast FPC diagnosis, year of breast FPC diagnosis (single year for 1157 SPCs, and grouped years for 5 cases, as insufficient matches were made using a single year) and being alive when the corresponding SPC was diagnosed. ^f^The median (percentile 25—percentile 75) age at diagnosis of FPC was 60 (50–70) for metachronous SPC and 60 (50–70) for the corresponding FPC only patients

Individuals with SPCs were matched 1:3 to individuals without SPCs who were alive when the corresponding SPC was diagnosed, by five-year age group (from 15–19 to 70–74, and ≥ 75) at breast FPC diagnosis and year of breast FPC diagnosis (Fig. 1).

### Statistical analysis

For patients with a breast FPC who developed an SPC (FPC + SPC), survival time was considered as the time between SPC diagnosis and death from any cause or end of study period (30 June 2021), whichever occurred first. Survival time for patients with a breast FPC who did not develop an SPC (FPC only) was considered as the time between FPC diagnosis and death or end of study period, whichever occurred first, minus the time between the SPC diagnosis and the FPC diagnosis of the matched FPC + SPC patient.

Cox-proportional hazards regression analyses were used to compute hazard ratios (HRs) for all-cause mortality adjusted for age (continuous) at breast FPC diagnosis with the corresponding 95% confidence intervals (CIs). The proportional hazards assumption was evaluated using Schoenfeld residuals.

The observed cumulative mortality was estimated using 1 – Kaplan–Meier [25]. The relative risk (RR) and risk difference (RD) of observed cumulative mortality were estimated for the comparison between patients with FPC + SPC, and those with an FPC only.

Several sensitivity analyses were performed defining SPCs as diagnosed two and six months after the breast FPC, as well as between two and twelve months following the breast FPC.

Data analysis was conducted using the statistical program Stata (Version 15.1, StataCorp, College Station, Texas). Results were considered statistically significant for p-value < 0.05.

## Results

A total of 1162 patients with a breast FPC + SPC and 3486 patients with a breast FPC only were included (Fig. 1). The most common SPC sites were breast, digestive organs, thyroid, lung, and corpus uteri (Table 1). During follow-up, a total of 1242 deaths (35.6%) were reported among patients with a breast FPC only, whereas 697 (60.0%) deaths were reported among patients with an FPC + SPC. Overall, patients with a synchronous or metachronous SPC had a significantly higher hazard of death compared to patients with a breast FPC only [HR = 1.56 (95%CI 1.29–1.89) and HR = 2.85 (95%CI 2.56–3.17), respectively]. Statistically significant HRs were observed for lung, stomach, non-Hodgkin lymphoma and breast synchronous SPCs, with HRs ranging from 1.28 (95%CI 1.00–1.64) to 7.72 (95%CI 1.89–31.57). For patients diagnosed with a metachronous SPC, liver, stomach, ovary, lung, rectum, corpus uteri, colon, breast, and non-Hodgkin lymphoma had statistically significant HRs, with estimates ranging from 1.88 (95%CI 1.10–3.21) to 13.10 (95%CI 6.26–27.43).

**Table 1:** Observed deaths up to 30 June 2021 among first primary breast cancer patients and respective hazard ratios, according to site of synchronous or metachronous second primary cancer diagnosis

	Total N	Deaths N (%)	Adjusted HR^a^ (95%CI)
**Synchronous** **SPCs**			
FPC only	795	343 (43.1)	1
FPC + SPC^b^	265	153 (57.7)	**1.56 (1.29–1.89)**
Stomach	9	8 (88.9)	**7.28 (2.83–18.73)**
Colon	13	10 (76.9)	1.41 (0.66–3.00)
Lung	6	5 (83.33)	**7.72 (1.89–31.57)**
Breast	178	88 (49.4)	**1.28 (1.00–1.64)**
Corpus uteri	6	4 (66.7)	0.52 (0.11–2.31)
Kidney	5	1 (20.0)	0.49 (0.06–4.24)
Thyroid	9	4 (44.4)	1.21 (0.38–3.81)
Non-Hodgkin lymphoma	6	6 (100.0)	**2.97 (1.06–8.28)**
Other SPCs^c^	33	27 (81.8)	**2.57 (1.61–4.12)**
**Metachronous** **SPCs**			
FPC only	2691	899 (33.3)	1
FPC + SPC^b^	897	544 (60.6)	**2.85 (2.56–3.17)**
Stomach	85	67 (78.8)	**6.52 (4.63–9.19)**
Colon	110	64 (58.2)	**2.01 (1.49–2.71)**
Rectum	39	22 (56.4)	**2.59 (1.52–4.40)**
Liver	21	20 (95.2)	**13.10 (6.26–27.43)**
Lung	79	64 (81.1)	**6.01 (4.31–8.61)**
Breast	137	57 (41.6)	**1.97 (1.42–2.72)**
Cervix uteri	20	8 (40.0)	1.09 (0.49–2.43)
Corpus uteri	68	37 (54.4)	**2.21 (1.48–3.29)**
Ovary	23	18 (57.7)	**6.28 (3.16–12.45)**
Bladder	33	19 (57.6)	1.15 (0.68–1.93)
Thyroid	71	20 (28.2)	1.04 (0.63–1.74)
Non-Hodgkin lymphoma	38	21 (55.3)	**1.88 (1.10–3.21)**
Other SPCs^d^	173	127 (73.4)	**4.53 (3.58–5.74)**

*CI* confidence interval, *FPC* first primary cancer, *HR* hazard ratio, *SPC* second primary cancer

Only shown for synchronous SPC sites with at least five cases and metachronous SPC sites with at least 20 cases

^a^Adjusted for age (continuous) at breast FPC diagnosis

^b^Stomach (C16), Colon (C18), Rectum (C19-C20), Liver and intrahepatic bile ducts (C22), Lung (including trachea and bronchus, C33-C34), Breast (C50), Cervix uteri (C53), Corpus uteri (C54), Ovary (C56), Kidney (C64), Bladder (C67), Thyroid (C73), Non-Hodgkin lymphoma (C85) defined according to the International Statistical Classification of Diseases and Related Health Problems 10th Revision [23]

^c^Other synchronous SPC sites include: Lip, oral cavity and pharynx (C00-C14), Small intestine (C17), Rectum (C19-C20), Anus and anal canal (C21), Liver and intrahepatic bile ducts (C22), Gallbladder (C23), Pancreas (C25), Nasal cavity and middle ear (C30), Melanoma of skin (C43), Vulva (C51), Cervix uteri (C53), Uterus, part unspecified (C55), Ovary (C56), Renal pelvis (C65), Ureter (C66), Bladder (C67), Without specification of site (C80), Malignant immunoproliferative diseases (C88), Multiple myeloma and malignant plasma cell neoplasms (C90), Lymphoid leukaemia (C91), Myelodysplastic syndromes (D46) defined according to the International Statistical Classification of Diseases and Related Health Problems 10th Revision [23]

^d^Other synchronous SPC sites include: Lip, oral cavity and pharynx (C00-C14), Oesophagus (C15), Small intestine (C17), Anus and anal canal (C21), Gallbladder (C23), Pancreas (C25), Nasal cavity and middle ear (C30), Larynx (C32), Thymus (C37), Bone and articular cartilage of limbs (C40), Melanoma of skin (C43), Mesothelioma (C45), Kaposi sarcoma (C46), Peripheral nerves and autonomic nervous system (C47), Vulva (C51), Vagina (C52), Uterus, part unspecified (C55), Kidney (C64), Meninges (C70), Adrenal gland (C74), Without specification of site (C80), Hodgkin lymphoma (C81), Malignant immunoproliferative diseases (C88), Multiple myeloma and malignant plasma cell neoplasms (C90), Lymphoid leukaemia (C91), Myeloid leukaemia (C92-C94), Leukaemia of unspecified cell type (C95), Myelodysplastic syndromes (D46), Other neoplasms of uncertain or unknown behaviour of lymphoid, haematopoietic and related tissue (D47) defined according to the International Statistical Classification of Diseases and Related Health Problems 10th Revision [23]

**Fig. 2:**
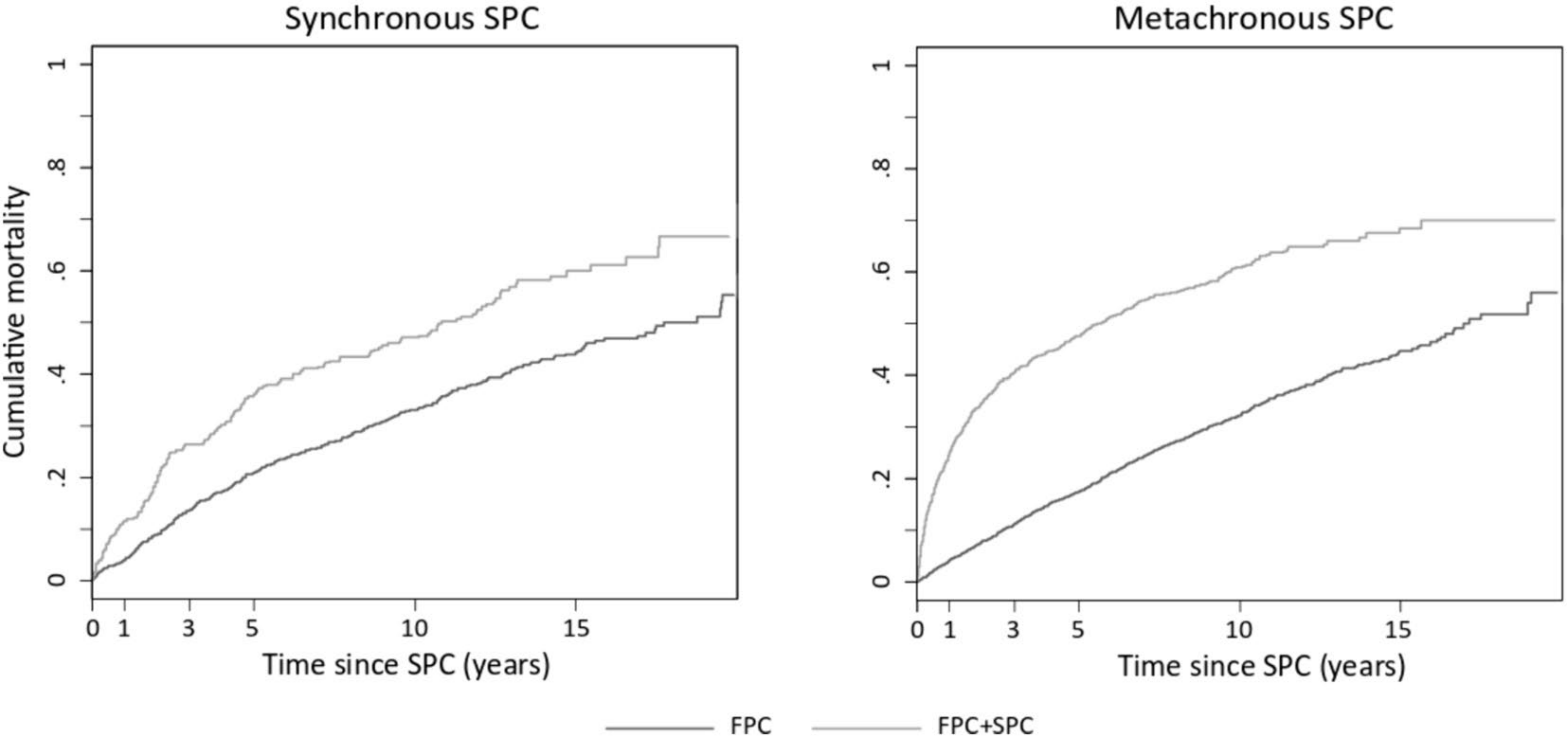
Observed cumulative mortality^a^ of breast first primary cancer patients with and without a synchronous or metachronous second primary cancer diagnosis. *FPC* first primary cancer, *SPC* second primary cancer. ^a^Calculated using 1 – Kaplan–Meier [25]

The cumulative mortality of patients with a breast FPC only and those with an FPC + SPC is shown in Table 2 and Fig. 2. The 15-year cumulative mortality was 59.5% (95%CI 57.6–61.45) for patients with an FPC + synchronous SPC and 43.6% (95%CI 41.7–45.5) for patients with a breast FPC only, with an RD of 15.9%. A gradual decrease in the RR from 2.79 at one-year to 1.36 at 15-years was observed. The cumulative mortality at 15-years was 68.7% (95%CI 66.7–70.6) for patients with an FPC + metachronous SPC versus 44.8% (95%CI 42.9–46.7) for patients with a breast FPC only, with an RD of 23.9%. The RR ranged from 5.81 at one-year to 1.53 at 15-years.

**Table 2:** Observed cumulative mortality^a^ of first primary breast cancer patients with and without a synchronous or metachronous second primary cancer diagnosis

	FPC only	FPC + SPC	RR^b^	RD^c^ %
Time since SPC, years	Cumulative mortality % (95%CI)	Cumulative mortality % (95%CI)		
**Synchronous** **SPCs**				
1	3.9 (3.1–4.7)	10.9 (10.1–11.7)	2.79	7.00
3	13.6 (12.5–14.7)	25.7 (24.5–26.8)	1.89	12.10
5	20.7 (19.5–22.0)	35.1 (33.9–36.3)	1.70	14.40
10	33.1 (31.7–34.6)	46.4 (44.9–47.9)	1.40	13.30
15	43.6 (41.7–45.5)	59.5 (57.6–61.45)	1.36	15.90
**Metachronous** **SPC**s				
1	4.3 (3.5–5.1)	25.0 (24.2–25.9)	5.81	20.70
3	11.3 (10.2–12.4)	40.8 (39.7–41.9)	3.61	29.50
5	17.5 (16.2–18.7)	47.7 (46.5–49.0)	2.73	30.20
10	32.3 (30.8–33.8)	61.1 (59.6–62.5)	1.89	28.80
15	44.8 (42.9–46.7)	68.7 (66.7–70.6)	1.53	23.90

*CI* confidence interval, *FPC* first primary cancer, *RD* risk difference, *RR* relative risk, *SPC* second primary cancer

^a^Calculated using 1 – Kaplan–Meier [25]

^b^Calculated as observed mortality in FPC + SPC / observed mortality in FPC only

^c^Calculated as observed mortality FPC + SPC – observed mortality in FPC only

Analyses defining synchronous SPCs as occurring within two and six months of a breast FPC generally resulted in lower HR estimates (Supplementary Table 1). The HR was 1.46 (95%CI 1.16–1.83) for synchronous SPCs diagnosed within two months of the breast FPC, 1.84 (95%CI 1.29–2.64) for SPCs diagnosed between two and 12 months after the breast FPC, and 2.71 (95%CI 2.45–3.02) for metachronous SPCs diagnosed more than 12 months after the breast FPC. When using the cut-off of six months to define synchronous and metachronous SPCs, the HRs were 1.51 (95%CI 1.23–1.86) for synchronous SPCs and 2.77 (95%CI 2.49–3.07) for metachronous SPCs.

The sensitivity analyses for cumulative mortality, using the two- or six-months cut-off to define synchronous and metachronous SPCs, yielded lower estimates for patients diagnosed with an SPC within two months of the breast FPC. No differences in cumulative mortality were observed for metachronous SPCs using the different time cut-offs (Supplementary Table 2).

## Discussion

Our population-based cohort study showed that patients with a breast FPC diagnosed with an SPC had a higher hazard of death of 1.5-fold for synchronous SPCs and almost three-fold for metachronous SPCs compared with patients with a breast FPC only. The sites with higher HRs were similar for synchronous and metachronous SPCs, namely lung, stomach, breast, non-Hodgkin lymphoma, liver, genital, and colon and rectum. Overall, the cumulative mortality estimates were higher for patients with a synchronous or metachronous SPC compared to patients with a breast FPC only.

Previous research has described a higher mortality among patients with an SPC compared to patients with a breast FPC only [11–14]. Deng and colleagues published data from a large cohort including over 60 thousand women with breast cancer from the USA, using the Surveillance, Epidemiology, and End Results, diagnosed between 2000 to 2016, with an average follow-up time of 42 months [14]. They found that patients with any SPC diagnosed more than six months after the FPC had an HR of death of 1.18 compared to patients with a breast cancer only. The SPC sites with higher HRs were breast, lung, colon, rectum, uterus, lymphoma, melanoma, thyroid, and leukemia [14], which is in line with our findings. Their 15-year cumulative mortality estimates were 67.5% for patients with an SPC and 60.4% for patients with an FPC only [14]. Many previous studies have considered breast cancer as the FPC and/or SPC [12, 13, 26]. A systematic review by Pan and colleagues included 15 relevant studies, with patients with unilateral breast cancer only and patients with bilateral breast cancer. They compared the HRs of death among those with a unilateral breast cancer and a bilateral breast cancer, combining different definitions for synchronous and a metachronous within three, six and 12 months as the cut-off time. Compared to patients with a unilateral breast cancer only, they estimated an HR of 1.68 for bilateral breast cancer, 2.01 for synchronous breast SPC and 3.22 for a metachronous breast SPC [12]. Using data from the Danish Breast Cancer Cooperative Group database, Langballe and colleagues studied nearly 70 thousand women with breast cancer diagnosed between 1978 and 2012, and followed until 2015 for cancer-specific death. Compared to patients with a unilateral breast FPC, those with a bilateral breast cancer had a two-fold higher hazard of death [13].

We found that patients with synchronous and metachronous SPCs both have a higher mortality compared to patients with a breast FPC only, though mortality was lower among those with synchronous SPCs. Patients diagnosed with synchronous SPCs may be under closer medical monitoring due to their breast FPC being more recently diagnosed. Consequently, there is a possibility that a subsequent SPC is detected at an earlier stage and given the closer medical surveillance, prompt initiation of treatment may contribute to their lower mortality compared to those with metachronous SPCs. Conversely, patients diagnosed with metachronous SPCs might be detected at later stages or be associated with more aggressive tumor characteristics, resulting in an even worse prognosis among these patients. A previous systematic review described above by Pan and colleagues estimated higher HR for synchronous breast SPC and metachronous breast SPC compared to patients with a unilateral breast cancer only [12]. Moreover, a study by Pacheco-Figueiredo and colleagues reported that the survival of patients with metachronous SPCs may be more influenced by the site of the SPC whereas in patients with synchronous SPCs, survival varied considering the site of the FPC [18]. In the current study, we may hypothesize that the breast FPC is contributing to the higher survival observed among patients with a synchronous SPC.

### Strengths and limitations

Our data was collected from a population-based registry, RORENO, which is highly representative of patients with breast cancer from Northern Portugal. It has a large catchment area, integrating public and private institutions involved in the diagnosis and care of patients with cancer; and has a long-term and complete follow-up with only 1% losses identified. Furthermore, we provide sensitivity analyses considering the cut-off for defining synchronous and metachronous SPCs at two and six months. However, some limitations should be discussed. Patients were followed for the occurrence of an SPC until 2015, which was the maximum data available from RORENO at the time of the study. This allowed a minimum follow-up of five years and a maximum follow-up of 15 years. Although we would likely have a larger number of patients with metachronous SPCs if there was a longer follow-up to identify multiple primary cancers, we do not believe that the results of our study would be any different. In the present work, we are assuming, that all other causes of death, besides cancer related death, influence the survival of patients with an FPC only or an FPC + SPC in a similar manner. However, HRs will inherently be influenced by the timing of SPC diagnoses relative to the FPC, while synchronous SPCs occur almost concurrently with the FPC. Nevertheless, our study considers the FPC and SPC by calculating survival time for all patients from the moment the SPC is diagnosed, irrespective of whether they had an SPC or not, to ensure that patients with FPCs only have at least the same duration of survival until the occurrence of the SPC of the matched patient. This is necessary because the effect measures will be influenced by the SPC time from the first primary to the second primary. Some site-specific HRs may lack the statistical power needed for robust interpretation on an individual basis, particularly for SPCs with small sample sizes or low event rates, these estimates should be interpreted with caution. Information on clinical data that could influence the prognosis of patients with a breast cancer was not available, namely stage at FPC and SPC diagnosis, hormonal receptor status of the tumor, treatment regime, as well as menopausal status, family history or genetic susceptibility. Previous studies have shown that lifestyle risk factors, such as obesity, tobacco, treatment side-effects, or even modified standard therapy may impact the survival of patients with multiple cancers [12, 14, 23, 27].

### Conclusion

This is the first comprehensive analysis in Northern Portugal evaluating the impact of breast cancer, which is the most incident cancer worldwide and in Portugal, on SPC survivorship. We found patients with an SPC following a breast FPC have a higher mortality than those with a breast FPC only and mortality was higher for those with a metachronous SPC. Lung, stomach, breast, non-Hodgkin lymphoma, liver, genital, and colon and rectum were SPC sites with the worst prognosis. These findings highlight the need for surveillance of patients with a breast cancer and management of the expectations of these patients.

### Supplementary Information

Below is the link to the electronic supplementary material.

Supplementary file1 (DOCX 34 kb)

## Abbreviations

95%CI: 95% Confidence interval
FPC: First primary cancer
HR: Hazards ratio
IACR: International Association of Cancer Registries
IARC: International Agency for Research on Cancer
RD: Risk difference
RR: Relative risk
RORENO: North Region Cancer Registry of Portugal
SPC: Second primary cancer
USA: United States of America

## Declarations

### Data availability

The data that support the findings of this study are available from the North Region Cancer Registry (RORENO), but restrictions apply to the availability of these data, which were used under license for the current study and so are not publicly available. Data may be available from the authors upon reasonable request and with permission of RORENO.
