# Supplementary material for: The contribution of second primary cancers to the mortality of patients with a first primary breast cancer
Source: Breast Cancer Res Treat. 2024 Jun 13;207(2):323–30. doi: 10.1007/s10549-024-07361-3 (PMC11297125; doi:10.1007/s10549-024-07361-3)
Supplement: Supplementary file 1 — Supplementary file1 (DOCX 34 kb) [file 10549_2024_7361_MOESM1_ESM.docx]

**The contribution of second primary cancers to the mortality of patients with a first primary breast cancer**

Elisabete Gonçalves, Filipa Fontes, Jéssica Rocha Rodrigues, Rita Calisto, Maria José Bento, Nuno Lunet, Samantha Morais

**Supplementary Table 1.** Observed deaths up to 30 June 2021 among first primary breast cancer patients and respective hazard ratios, according to site of synchronous or metachronous second primary cancer diagnosis considering different periods of cut-off to define second primary cancers.

|  |  |  | **Total**  **N** |  | **Deaths**  **N (%)** |  | **Adjusted HR^a^**  **(95%CI)** |
| --- | --- | --- | --- | --- | --- | --- | --- |
| **Two months** | | | | | | | |
| **Synchronous**  **SPCs**  (within two months  of the FPC) | FPC only |  | 600 |  | 250 (41.7) |  | 1 |
|  | FPC+SPC^b^ |  | 200 |  | 108 (54.0) |  | **1.46 (1.16-1.83)** |
|  | Stomach |  | 6 |  | 6 (100.0) |  | **161.76 (12.61-2075.71)** |
|  | Colon |  | 7 |  | 6 (85.7) |  | 1.67 (0.59-4.69) |
|  | Lung |  | 3 |  | 2 (66.7) |  | 3.15 (0.45-22.05) |
|  | Breast |  | 164 |  | 79 (48.2) |  | 1.25 (0.96-1.63) |
|  | Corpus uteri |  | 0 |  | -- |  | -- |
|  | Kidney |  | 1 |  | 0 (0.0) |  | -- |
|  | Thyroid |  | 2 |  | 1 (50.0) |  | 2.20 (0.13-38.52) |
|  | Non-Hodgkin lymphoma |  | 3 |  | 3 (100.0) |  | 3.71 (0.71-19.23) |
|  | Other SPCs |  | 14 |  | 11 (78.6) |  | **2.62 (1.23-1.10)** |
|  |  |  |  |  |  |  |  |
| **Metachronous**  **SPCs**  (between two and twelve  months after the FPC) | FPC only |  | 195 |  | 93 (47.7) |  | 1 |
|  | FPC+SPC^b^ |  | 65 |  | 45 (69.2) |  | **1.84 (1.29-2.64)** |
|  | Stomach |  | 3 |  | 2 (66.7) |  | 4.27 (0.64-28.54) |
|  | Colon |  | 6 |  | 4 (66.7) |  | 1.78 (0.53-5.95) |
|  | Rectum |  | 1 |  | 1 (100.0) |  | -- |
|  | Liver |  | 0 |  | -- |  | -- |
|  | Lung |  | 3 |  | 3 (100.0) |  | -- |
|  | Breast |  | 14 |  | 9 (64.3) |  | 1.80 (0.81-3.99) |
|  | Cervix uteri |  | 1 |  | 0 (0.0) |  | -- |
|  | Corpus uteri |  | 6 |  | 4 (66.7) |  | 0.51 (0.11-2.31) |
|  | Ovary |  | 1 |  | 1 (100.0) |  | -- |
|  | Bladder |  | 1 |  | 1 (100.0) |  | -- |
|  | Thyroid |  | 7 |  | 3 (42.9) |  | 1.17 (0.31-4.47) |
|  | Non-Hodgkin lymphoma |  | 3 |  | 3 (100.0) |  | 6.87 (0.51-93.01) |
|  | Other SPCs |  | 19 |  | 14 (73.7) |  | **2.41 (1.25-4.61)** |
|  |  |  |  |  |  |  |  |
| **Metachronous**  **SPCs**  (more than two months  after the FPC) | FPC only |  | 2886 |  | 992 (34.4) |  | 1 |
|  | FPC+SPC^b^ |  | 962 |  | 589 (61.2) |  | **2.71 (2.45-3.02)** |
|  | Stomach |  | 88 |  | 69 (78.4) |  | **5.83 (4.20-8.10)** |
|  | Colon |  | 116 |  | 68 (58.6) |  | **1.91 (1.43-2.55)** |
|  | Rectum |  | 40 |  | 23 (57.5) |  | **2.65 (1.57-4.45)** |
|  | Liver |  | 21 |  | 20 (95.2) |  | **13.10 (6.26-27.43)** |
|  | Lung |  | 82 |  | 67 (81.7) |  | **6.17 (4.39-8.68)** |
|  | Breast |  | 151 |  | 66 (43.7) |  | **1.93 (1.43-2.60)** |
|  | Cervix uteri |  | 21 |  | 8 (38.1) |  | 1.07 (0.48-2.40) |
|  | Corpus uteri |  | 74 |  | 41 (55.4) |  | **1.93 (1.32-2.83)** |
|  | Ovary |  | 24 |  | 19 (79.2) |  | **6.63 (3.39-12.96)** |
|  | Bladder |  | 33 |  | 19 (79.2) |  | 1.15 (0.68-1.93) |
|  | Thyroid |  | 78 |  | 23 (29.5) |  | 1.06 (0.66-1.71) |
|  | Non-Hodgkin lymphoma |  | 41 |  | 24 (58.5) |  | **2.00 (1.21-3.29)** |
|  | Other SPCs |  | 193 |  | 142 (73.4) |  | **3.98 (3.19-4.95)** |
|  |  |  |  |  |  |  |  |
| **Six months** | | | | | | | |
| **Synchronous**  **SPCs**  (within six months  of the FPC) | FPC only |  | 690 |  | 300 (43.5) |  | 1 |
|  | FPC+SPC^b^ |  | 230 |  | 129 (56.1) |  | **1.51 (1.23-1.86)** |
|  | Stomach |  | 7 |  | 6 (85.7) |  | **6.39 (2.17-18.78)** |
|  | Colon |  | 11 |  | 10 (90.9) |  | 1.97 (0.90-4.32) |
|  | Lung |  | 3 |  | 2 (66.7) |  | 3.14 (0.45-22.05) |
|  | Breast |  | 172 |  | 84 (48.8) |  | 1.25 (0.97-1.62) |
|  | Corpus uteri |  | 0 |  | -- |  | -- |
|  | Kidney |  | 4 |  | 1 (25.0) |  | 0.62 (0.07-5.57) |
|  | Thyroid |  | 4 |  | 1 (25.0) |  | 0.93 (0.10-8.79) |
|  | Non-Hodgkin lymphoma |  | 6 |  | 6 (100.0) |  | **2.97 (1.06-8.28)** |
|  | Other SPCs |  | 23 |  | 19 (82.6) |  | **2.83 (1.61-4.96)** |
|  |  |  |  |  |  |  |  |
| **Metachronous**  **SPCs**  (more than six months  after the FPC) | FPC only |  |  |  |  |  | 1 |
|  | FPC+SPC^b^ |  | 932 |  | 568 (60.9) |  | **2.77 (2.49-3.07)** |
|  | Stomach |  | 87 |  | 69 (79.3) |  | **6.58 (4.70-9.22)** |
|  | Colon |  | 112 |  | 64 (57.1) |  | **1.88 (1.39-2.53)** |
|  | Rectum |  | 40 |  | 23 (57.5) |  | **2.65 (1.57-4.45)** |
|  | Liver |  | 21 |  | 20 (95.2) |  | **13.10 (6.26-27.43)** |
|  | Lung |  | 82 |  | 67 (81.7) |  | **6.17 (4.39-8.68)** |
|  | Breast |  | 143 |  | 61 (42.7) |  | **1.98 (1.44-2.70)** |
|  | Cervix uteri |  | 21 |  | 8 (38.1) |  | 1.07 (0.48-2.40) |
|  | Corpus uteri |  | 74 |  | 41 (55.4) |  | **1.93 (1.32-2.83)** |
|  | Ovary |  | 23 |  | 18 (78.3) |  | **6.28 (3.16-12.45)** |
|  | Bladder |  | 33 |  | 19 (57.6) |  | 1.15 (0.68-1.93) |
|  | Thyroid |  | 76 |  | 23 (30.3) |  | 1.09 (0.68-1.76) |
|  | Non-Hodgkin lymphoma |  | 38 |  | 21 (55.3) |  | **1.88 (1.10-3.21)** |
|  | Other SPCs |  | 182 |  | 134 (73.6) |  | **4.24 (3.37-5.32)** |

CI, confidence interval; FPC, first primary cancer; HR, hazard ratio; NA, not applicable; SPC, second primary cancer.

^a^ Adjusted for age (continuous) at FPC diagnosis.

^b^ Stomach (C16), Colon (C18), Rectum (C19-C20), Liver and intrahepatic bile ducts (C22), Lung (including trachea and bronchus, C33-C34), Breast (C50), Cervix uteri (C53), Corpus uteri (C54), Ovary (C56), Kidney (C64), Bladder (C67), Thyroid (C73), Non-Hodgkin lymphoma (C85) defined according to the International Statistical Classification of Diseases and Related Health Problems 10th Revision [23].

**Supplementary Table 2.** Observed cumulative mortality^a^ of first primary breast cancer patients with and without a synchronous or metachronous second primary cancer diagnosis considering different periods of cut-off to define second primary cancers.

|  |  |  | **FPC only** |  | **FPC+SPC** |  | RR^b^ |  | RD^c^ % |
| --- | --- | --- | --- | --- | --- | --- | --- | --- | --- |
|  | **Time since SPC,**  **years** |  | Cumulative mortality  % (95%CI) |  | Cumulative mortality  % (95%CI) |  |  |  |  |
| **Two months** | | | | | | | | | |
| **Synchronous**  **SPCs**  (within two months  of the FPC) | 1 |  | 3.8 (3.0-4.6) |  | 8.5 (7.8-9.3) |  | 2.24 |  | 4.70 |
|  | 3 |  | 14.0 (12.9-15.1) |  | 21.5 (20.4-22.6-22.6) |  | 1.54 |  | 7.50 |
|  | 5 |  | 20.5 (19.3-21.7) |  | 30.5 (29.3-31.7) |  | 1.49 |  | 10.00 |
|  | 10 |  | 31.8 (30.35-33.3) |  | 42.5 (41.0-44.0) |  | 1.34 |  | 10.70 |
|  | 15 |  | 41.58 (39.7-43.51) |  | 55.1 (53.2-57.1) |  | 1.33 |  | 13.60 |
|  |  |  |  |  |  |  |  |  |  |
| **Metachronous**  **SPCs**  (between two and twelve  months after the FPC) | 1 |  | 4.1 (3.3-4.9) |  | 18.5 (17.7-19.2) |  | 4.51 |  | 14.40 |
|  | 3 |  | 12.3 (11.2-13.4) |  | 38.5 (37.4-39.6) |  | 3.13 |  | 26.20 |
|  | 5 |  | 21.5 (20.3-22.8) |  | 49.2 (48.0-50.5) |  | 2.29 |  | 27.70 |
|  | 10 |  | 36.9 (35.5-38.4) |  | 58.5 (57.0-59.9) |  | 1.59 |  | 21.60 |
|  | 15 |  | 50.5 (48.7-52.4) |  | 73.1 (71.3-75.0) |  | 1.45 |  | 22.60 |
|  |  |  |  |  |  |  |  |  |  |
| **Metachronous**  **SPCs**  (more than two months  after the FPC) | 1 |  | 4.3 (3.5-5.1) |  | 24.6 (23.8-25.4) |  | 5.72 |  | 20.30 |
|  | 3 |  | 11.4 (10.3-12.5) |  | 40.6 (39.5-41.7) |  | 3.56 |  | 29.20 |
|  | 5 |  | 17.7 (16.5-19.0) |  | 47.8 (46.6-49.1) |  | 2.70 |  | 30.10 |
|  | 10 |  | 32.6 (31.2-34.1) |  | 60.8 (59.3-62.3) |  | 1.87 |  | 28.20 |
|  | 15 |  | 45.3 (43.4-47.3) |  | 69.7 (67.8-71.7) |  | 1.54 |  | 24.40 |
|  |  |  |  |  |  |  |  |  |  |
| **Six months** | | | | | | | | | |
| **Synchronous**  **SPCs**  (within six months  of the FPC) | 1 |  | 4.1 (3.2-4.9) |  | 10.4 (9.7-11.2) |  | 2.54 |  | 6.30 |
|  | 3 |  | 14.1 (12.9-15.2) |  | 24.3 (23.2-25.4) |  | 1.72 |  | 10.20 |
|  | 5 |  | 20.7 (19.5-22.0) |  | 33.9 (32.7-35.1) |  | 1.64 |  | 13.20 |
|  | 10 |  | 33.2 (31.7-34.7) |  | 46.1 (44.6-47.5) |  | 1.39 |  | 12.90 |
|  | 15 |  | 43.6 (41.7-45.5) |  | 57.4 (55.5-59.3) |  | 1.32 |  | 13.80 |
|  |  |  |  |  |  |  |  |  |  |
| **Metachronous**  **SPCs**  (more than six months  after the FPC) | 1 |  | 4.2 (3.4-5.0) |  | 24.6 (23.8-25.4) |  | 5.86 |  | 20.40 |
|  | 3 |  | 11.3 (10.2-12.4) |  | 40.5 (39.4-41.6) |  | 3.58 |  | 29.20 |
|  | 5 |  | 17.6 (16.4-18.8) |  | 47.6 (46.3-48.8) |  | 2.70 |  | 30.00 |
|  | 10 |  | 32.3 (30.8-33.7) |  | 60.5 (59.0-62.0) |  | 1.87 |  | 28.20 |
|  | 15 |  | 44.7 (42.7-46.6) |  | 69.8 (67.9-71.8) |  | 1.56 |  | 25.10 |

CI, confidence interval; FPC, first primary cancer; RD, risk difference; RR, relative risk; SPC, second primary cancer.

^a^ Calculated using 1 – Kaplan-Meier [25].

^b^ Calculated as observed mortality in FPC+SPC / observed mortality in FPC only.

^c^ Calculate as observed mortality FPC+SPC – observed mortality in FPC only.
